# XGBoost Classifier Based on Computed Tomography Radiomics for Prediction of Tumor-Infiltrating CD8^+^ T-Cells in Patients With Pancreatic Ductal Adenocarcinoma

**DOI:** 10.3389/fonc.2021.671333

**Published:** 2021-05-19

**Authors:** Jing Li, Zhang Shi, Fang Liu, Xu Fang, Kai Cao, Yinghao Meng, Hao Zhang, Jieyu Yu, Xiaochen Feng, Qi Li, Yanfang Liu, Li Wang, Hui Jiang, Jianping Lu, Chengwei Shao, Yun Bian

**Affiliations:** ^1^ Department of Radiology, Changhai Hospital, Navy Medical University, Shanghai, China; ^2^ Department of Pathology, Changhai Hospital, Navy Medical University, Shanghai, China

**Keywords:** pancreatic ductal adenocarcinoma, CD8 positive T lymphocytes, contrast-enhanced computed tomography images, radiomics, prognosis

## Abstract

**Objectives:**

This study constructed and validated a machine learning model to predict CD8^+^ tumor-infiltrating lymphocyte expression levels in patients with pancreatic ductal adenocarcinoma (PDAC) using computed tomography (CT) radiomic features.

**Materials and Methods:**

In this retrospective study, 184 PDAC patients were randomly assigned to a training dataset (n =137) and validation dataset (n =47). All patients were divided into CD8^+^ T-high and -low groups using X-tile plots. A total of 1409 radiomics features were extracted from the segmentation of regions of interest, based on preoperative CT images of each patient. The LASSO algorithm was applied to reduce the dimensionality of the data and select features. The extreme gradient boosting classifier (XGBoost) was developed using a training set consisting of 137 consecutive patients admitted between January 2017 and December 2017. The model was validated in 47 consecutive patients admitted between January 2018 and April 2018. The performance of the XGBoost classifier was determined by its discriminative ability, calibration, and clinical usefulness.

**Results:**

The cut-off value of the CD8^+^ T-cell level was 18.69%, as determined by the X-tile program. A Kaplan−Meier analysis indicated a correlation between higher CD8^+^ T-cell levels and better overall survival (*p* = 0.001). The XGBoost classifier showed good discrimination in the training set (area under curve [AUC], 0.75; 95% confidence interval [CI]: 0.67–0.83) and validation set (AUC, 0.67; 95% CI: 0.51–0.83). Moreover, it showed a good calibration. The sensitivity, specificity, accuracy, positive and negative predictive values were 80.65%, 60.00%, 0.69, 0.63, and 0.79, respectively, for the training set, and 80.95%, 57.69%, 0.68, 0.61, and 0.79, respectively, for the validation set.

**Conclusions:**

We developed a CT-based XGBoost classifier to extrapolate the infiltration levels of CD8^+^ T-cells in patients with PDAC. This method could be useful in identifying potential patients who can benefit from immunotherapies.

## Introduction

The microenvironment of pancreatic ductal adenocarcinoma (PDAC) is highly immunosuppressive and heterogeneous, characterized by an abundant desmoplastic stroma, inflammatory response, and neovascularization (1). Even with surgical resection, the radical resection rate is only approximately 18% (2), and the prognosis remains poor (3). Traditional chemotherapy is minimally effective, despite some recent success (4).

Tumors are a proliferation of abnormal cells that can escape immune eradication (5). The occurrence of immune escape is a key process in cancer progression. Immunotherapy, which aims to stimulate the body’s immune system against tumor cells, can overcome this problem. The recent success of immunotherapy targeting immune checkpoint inhibitors (ICI), such as the programmed cell death protein 1 (PD1) and PD1 ligand (PD-L1) pathways, has shed new light on the treatment of patients with tumors (6, 7). Nonetheless, treatment with these drugs has failed to show significant clinical benefit in unselected patients with PDAC, whose objective response rate to ICI therapy has been approximately 5% in previous clinical trials (8, 9). Therefore, there is a clear need to develop related predictive biomarkers to identify subsets of patients who may benefit from ICI therapy. An effective ICI therapy prerequisite is a high level of CD8^+^ tumor-infiltrating lymphocytes (TILs) in the tumor tissues, suggesting the importance of investigating CD8^+^ TILs (10). Immunohistochemistry is the gold standard for evaluating CD8^+^ TILs. However, the clinical application of immunohistochemistry is limited by its invasiveness, time consumption, tumor heterogeneity, and unrepeatability. In recent years, liquid biopsy is a hot spot of research. As a rapid and noninvasive alternative to tissue biopsy, liquid biopsy can capture circulating leukocytes to reflect cancer immunity (11). In general, cancer immunity consists of the local immunity in the tumor microenvironment and the systemic immunity in circulating peripheral blood (12). However, it is unclear whether systemic immune response always correlates with local immune response (12, 13). Takahiro Tsujikawa et al. have emphasized the utility of local immune monitoring for patient stratification, which could improve immunotherapy’s success rate (14).

Computed tomography (CT) is widely used for tumor detection, staging, and treatment response monitoring in clinical practice. Recently, radiomic biomarkers have been of great interest. They may extract spatial and temporal features from images that are useful in predicting the underlying molecular mechanisms, the tumor-immune microenvironment, and clinical outcome. Studies dealing with glioma, esophagus, lung, and liver cancers have shown that several imaging features extracted by radiomics were closely related to CD8^+^ TIL density (15–19). While reports are predicting clinicopathological results from tissue sections in PDAC (20–22), so far, there are no radiomic studies revealing the immune environment in PDAC. Subtyping of the immune microenvironment in PDAC will help design personalized immunotherapy for patients with PDAC.

Thus, we aimed to develop and validate a radiomic signature of immune infiltration in PDAC using radiomic data extracted from contrast-enhanced CT images in this study, which might help us identify the novel predictors of immunotherapy efficacy.

## Materials and Methods

### Patients

**Figure 1:**
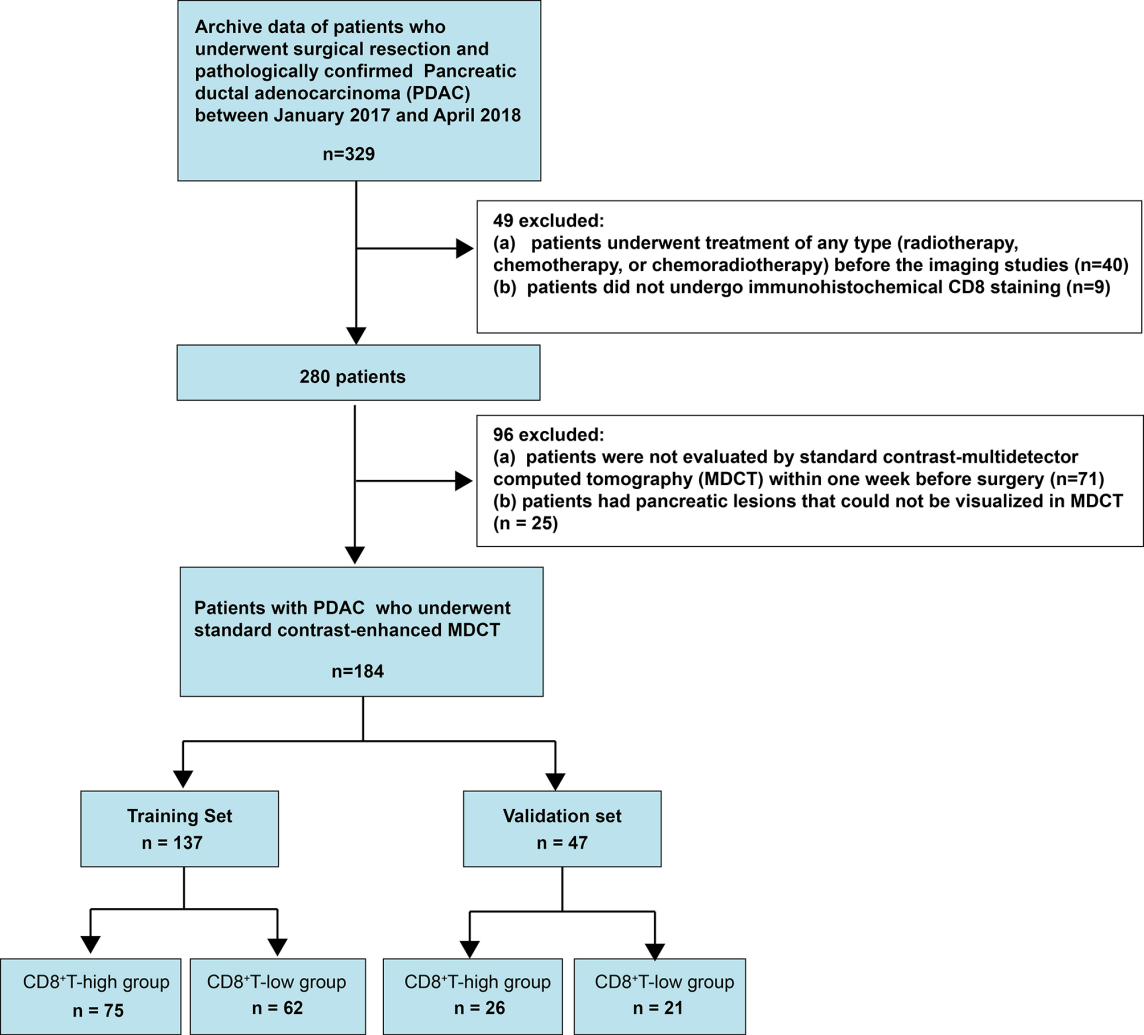
Flow chart visualizing the patient selection process.

This retrospective single-center cross-sectional study was reviewed and approved by the Biomedical Research Ethics Committee of our institution. The requirement for informed consent was waived by the Institutional Review Board. Data were obtained from consecutive patients treated for pancreatic cancer at our institution between January 2017 and April 2018 (**Figure 1**).

We included patients who (1) had undergone surgical treatment and (2) had pathologically confirmed PDAC. We excluded patients who (1) had undergone treatment of any type (radiotherapy, chemotherapy, or chemoradiotherapy) before the imaging studies, (2) did not undergo immunohistochemical CD8^+^ staining, (3) were not evaluated by contrast-enhanced multidetector computed tomography (MDCT) within 1 week preoperatively, or (4) had pancreatic lesions that could not be visualized on MDCT. Consequently, 184 consecutive patients with PDAC, including 120 men (age: 60.75 ± 10.31 years; range: 27–81 years) and 64 women (age: 63.11 ± 7.99 years; range: 37–80 years), were included. The prediction model was developed for a primary set that consisted of 137 consecutive patients, including 93 men (age: 60.44 ± 10.16 years; range: 27–80 years) and 44 women (age: 63.32 ± 7.96 years; range: 37–80 years), admitted between January 2017 and December 2017. Thus, 47 consecutive patients, including 27 men (age: 61.81 ± 10.94 years; range: 42–81 years) and 20 women (age: 62.65 ± 8.25 years; range: 42–71 years), admitted between January 2018 and April 2018, constituted an independent validation set.

### CT Scanning

Multiphasic CT was performed with a pancreas-specific protocol using 320-slice multidetector-row CT scanners (Aquilion ONE, Canon Medical Systems, Tokyo, Japan). The details are shown in **Appendix 1**.

### Pathological Image Analysis

All specimens were analyzed by two pathologists, one with 30 and the other with 20 years of experience in pancreatic pathology. Pathological examination and analysis were standardized as described previously (23). A CD8 antibody (DakoCytomation, Glostrup, Denmark) was used in pathological examinations. Each CD8-stained section was converted to digital pathological images by the scanner (NanoZoomer S60, Hamamatsu Healthcare, Japanese). The tumor boundaries were manually delineated, after which a customizable digital microscopy analysis platform (Visiopharm, Hørsholm, Denmark) was used to quantify CD8 in the tumor. The two pathologists examined the results, and the outcomes were determined by consensus. Subsequently, the proportion of the area of CD8 was calculated in the tumor. All pathologic results for the following factors were recorded: (1) T and N stages, which were evaluated based on the American Joint Committee on Cancer TNM Staging Manual, 8th Edition (24); (2) grade of differentiation; (3) duodenal invasion; (4) common bile duct invasion; (5) lymphovascular space invasion (LVSI); and (6) peripancreatic nerve.

### Radiological Imaging Analysis

The details are shown in **Appendix 1**.

### Radiomics Workflow

**Figure 2:**
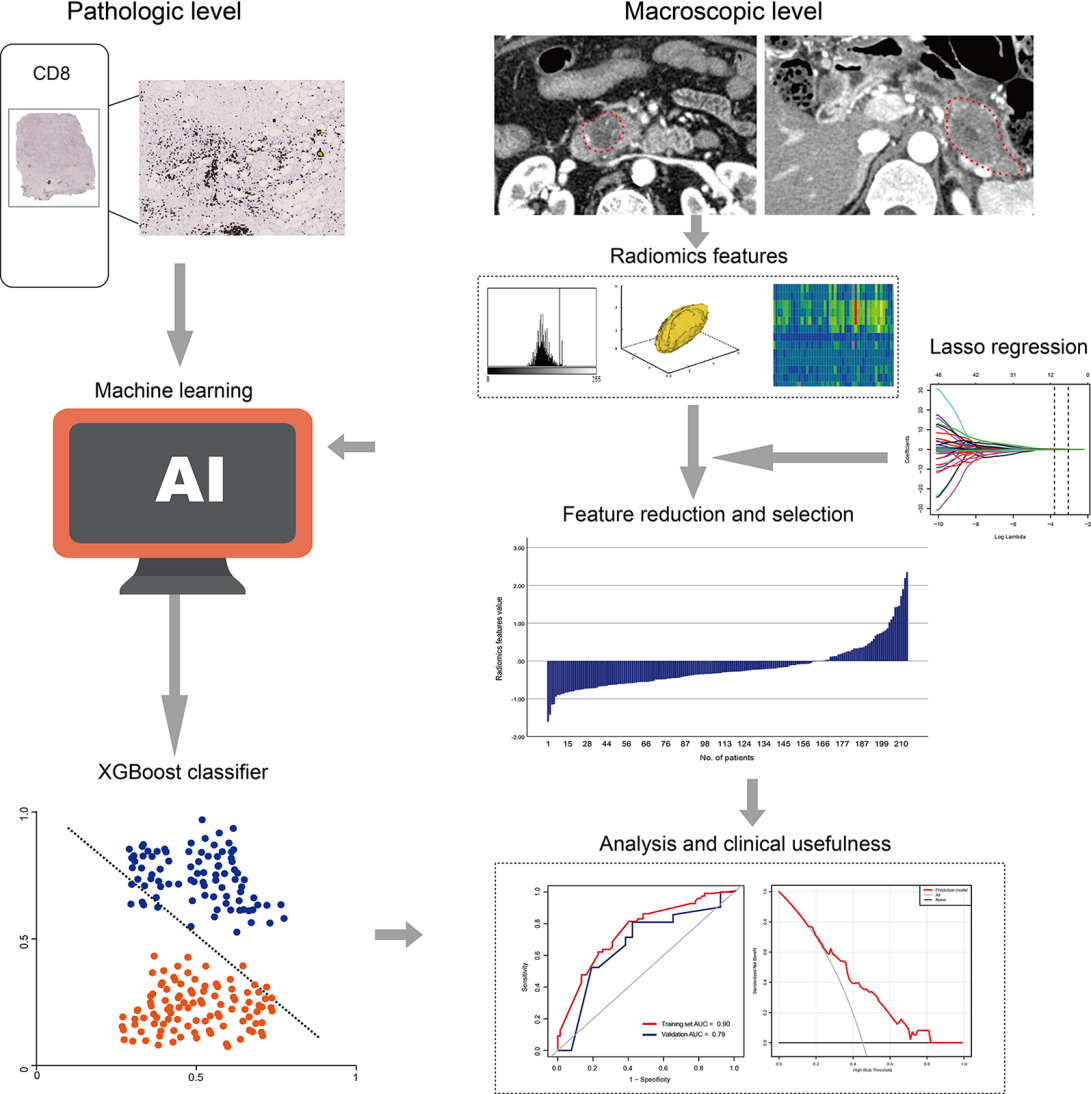
Radiomics workflow.

The radiomics workflow included: (1) image segmentation, (2) feature extraction, and (3) feature reduction and selection. The detailed method is shown in **Figure 2**.

We used the draw tool, which is available in the Editor module of 3D Slicer version 4.8.1 (open source software; https://www.slicer.org/), to delineate the tumors in multiple slices. The details are shown in **Appendix 1**.

To assess interobserver reliability, ROI segmentation was performed in a blinded fashion by two radiologists (readers 1 and 2, respectively). To evaluate intraobserver reliability, reader 1 repeated the feature extraction twice during a week period. This reader completed the remaining image segmentations, and the readout sessions were conducted over 2 weeks period. Assessments of interobserver and intraobserver reliability were performed by obtaining the intraclass correlation coefficient (ICC). ICC values >0.75 were selected for subsequent investigation.

### Statistical Analyses

Normal distribution and variance homogeneity tests were performed on all continuous variables. Those with normal distribution were expressed as mean and standard deviation, while those with non-normal distributions were expressed as medians and ranges. We evaluated the overall survival (OS). Deaths were set as events, and deaths attributed to other causes were set as censored observations. Survival times were calculated from surgery date to the time of death or the end of follow-up (August 1, 2020). First, the optimal cut-off CD8 level was determined with the help of X-tile (25). The X-tile program divided the patients into CD8-low and CD8-high groups, according to the optimal cut-off value. Kaplan−Meier estimates were applied to graph the survival curves, and the log-rank test was performed to analyze the differences between the curves. Second, we examined the differences in all variables between the CD8-low and CD8-high groups. Student’s t-test (normal distribution), Kruskal−Wallis H test (skewed distribution), and the chi-square test (categorical variables) were used to determine the intergroup statistical differences. Third, univariate regression analysis was applied to estimate the effect size between all variables and the CD8 groups. Fourth, the prediction model was constructed using an extreme gradient boosting classifier (XGBoost). XGBoost was performed using R software supplemented with the XGBoost package. The discrimination of the model was evaluated using a receiver operating characteristic (ROC) curve. The area under the curve (AUC) was calculated concurrently. The calibration of the model was assessed using the calibration curves and Hosmer−Lemeshow test. Finally, the model’s clinical usefulness was tested with a decision-curve analysis (DCA) by quantifying the net benefit at different threshold probabilities.

A two-tailed *p*-value <0.05 was considered statistically significant. All analyses were performed using R software (version 3.3.3, The R Foundation for Statistical Computing, Vienna, Austria).

## Results

### Clinical Characteristics

**Figure 3:**
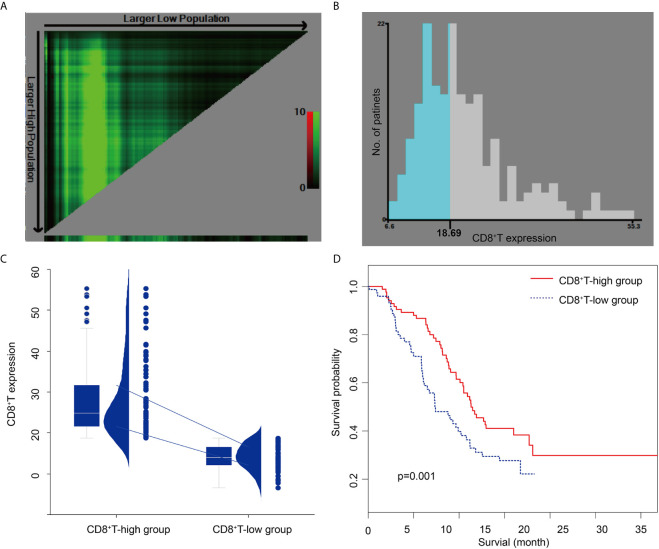
X-tile analysis of survival data in patients with pancreatic ductal adenocarcinoma **(A, B)** The optimal cut-off CD8^+^ T-cell level of 18.69%, determined by X-tile, is used to define the CD8^+^ T-high and CD8^+^ T-low groups. **(C)** CD8^+^ T in the CD8^+^ T-low group and the CD8^+^ T-high group. The chart includes a box plot, density plot, and dot plot. The 25th and 75th percentiles are shown as connecting lines between groups. **(D)** The Kaplan-Meier curve and log-rank test suggest that patients in the CD8^+^ T-high group survive significantly longer than those in the CD8^+^ T-low group.

Based on the optimal CD8 level cut-off determined by X-tile (18.69%; **Figures 3A, B**), all patients were divided into CD8-high (CD8 >18.69%, n = 101; 54.89%) and CD8-low (CD8 ≤18.69%, n = 83; 45.10%) groups (**Figure 3C**). CD8 expression was 28.07 ± 9.12% and 14.17 ± 2.93% in the CD8-high and CD8-low groups, respectively. Forty-six patients in the CD8-high group and 48 patients in the CD8-low group died. The Kaplan−Meier curves of the two groups were significantly distinct (*p =* 0.001). A log-rank test showed that the survival duration in the CD8-high group (22.63 months, 95% CI: 20.20–36.20) was significantly longer than that in the CD8-low group (14.67 months, 95% CI: 12.13–22.37) (**Figure 3D**). Among the clinical, pathological, and imaging characteristics that we investigated, T and N stage in the training set differed significantly between the two groups. The patient characteristics are shown in **Table 1**.

**Table 1:** Baseline characteristics of patients with pancreatic cancer.

Characteristics	Training set			Validation set		
	CD8^+^T-high (n=75)	CD8^+^T -low (n=62)	P-value	CD8^+^T -high (n=26)	CD8^+^T -low (n=21)	P-value
**Clinical characteristics**						
Sex, n (%)			0.48			0.97
Male	49 (65.33)	44 (70.97)		15 (57.69)	12 (57.14)	
Female	26 (34.67)	18 (29.03)		11 (42.31)	9 (42.86)	
Age, years (mean ± SD)	61.20 ± 9.96	61.56 ± 9.16	0.83	61.85 ± 9.78	62.57 ± 10.04	0.80
BMI, kg/m^2^ (mean ± SD)	22.98 ± 2.81	23.16 ± 2.88	0.71	94.55 ± 369.17	23.10 ± 2.40	0.38
Operation, n (%)			0.12			0.48
Pancreaticoduodenectomy	41 (54.67)	42 (67.74)		16 (61.54)	15 (71.43)	
Distal pancreatectomy	34 (45.33)	20 (32.26)		10 (38.46)	6 (28.57)	
**Pathological characteristics**						
T stage, n (%)			0.007			0.12
T1	3 (4.00)	7 (11.29)		0	3 (14.29)	
T2	31 (41.33)	37 (59.68)		13 (50.00)	11 (52.38)	
T3-4	41 (54.67)	18 (29.03)		13 (50.00)	7 (33.33)	
N stage, n (%)			0.01			0.50
N0	33 (44.00)	23 (37.10)		12 (46.15)	7 (33.33)	
N1	26 (34.67)	35 (56.45)		9 (34.62)	7 (33.33)	
N2	16 (21.33)	4 (6.45)		5 (19.23)	7 (33.33)	
Grade of differentiation, n (%)			1.00			0.22
Well-moderately	53 (70.67)	44 (70.97)		17 (65.38)	10 (47.62)	
Poorly-undifferentiated	22 (29.33)	18 (29.03)		9 (34.62)	11 (52.38)	
Duodenum Invasion, n (%)			0.82			0.97
Negative	51 (68.00)	41 (66.13)		15 (57.69)	12 (57.14)	
Positive	24 (32.00)	21 (33.87)		11 (42.31)	9 (42.86)	
Bile Invasion, n (%)			0.29			0.13
Negative	49 (65.33)	35 (56.45)		18 (69.23)	10 (47.62)	
Positive	26 (34.67)	27 (43.55)		8 (30.77)	11 (52.38)	
LVSI n (%)			0.17			0.13
Negative	46 (61.33)	45 (72.58)		18 (69.23)	10 (47.62)	
Positive	29 (38.67)	17 (27.42)		8 (30.77)	11 (52.38)	
Perineural invasion, n (%)			0.73			1.00
Negative	5 (6.67)	3 (4.84)		2 (7.69)	1 (4.76)	
Positive	70 (93.33)	59 (95.16)		24 (92.31)	20 (95.24)	
**CT characteristics**						
Tumor size, cm (median, rang)	3.98 ± 1.72	3.44 ± 1.48	0.05	4.17 ± 1.73	3.24 ± 1.42	0.06
Location, n (%)			0.12			0.48
Head	41 (54.67)	42 (67.74)		16 (61.54)	15 (71.43)	
Body and tail	34 (45.33)	20 (32.26)		10 (38.46)	6 (28.57)	
Pancreatitis, n (%)			0.94			0.41
No	44 (58.67)	36 (58.06)		13 (50.00)	13 (61.90)	
Yes	31 (41.33)	26 (41.94)		13 (50.00)	8 (38.10)	
PD cutoff and dilation, n (%)			0.86			0.87
No	16 (21.33)	14 (22.58)		8 (30.77)	6 (28.57)	
Yes	59 (78.67)	48 (77.42)		18 (69.23)	15 (71.43)	
CBD cutoff and dilation, n (%)			0.60			0.72
No	48 (64.00)	37 (59.68)		15 (57.69)	11 (52.38)	
Yes	27 (36.00)	25 (40.32)		11 (42.31)	10 (47.62)	
Parenchymal atrophy, n (%)			0.30			0.92
No	32 (42.67)	32 (51.61)		12 (46.15)	10 (47.62)	
Yes	43 (57.33)	30 (48.39)		14 (53.85)	11 (52.38)	
Contour abnormality, n (%)			0.94			0.71
No	10 (13.33)	8 (12.90)		6 (23.08)	3 (14.29)	
Yes	65 (86.67)	54 (87.10)		20 (76.92)	18 (85.71)	
Cyst, n (%)			0.33			0.30
No	71 (94.67)	56 (90.32)		22 (84.62)	20 (95.24)	
Yes	4 (5.33)	6 (9.68)		4 (15.38)	1 (4.76)	
Vascular invasion, n (%)			0.47			0.63
No	54 (72.00)	48 (77.42)		19 (73.08)	14 (66.67)	
Yes	21 (28.00)	14 (22.58)		7 (26.92)	7 (33.33)	

BMI, body mass index; PD, pancreatic duct; CBD, common bile duct; LVSI, lymphvascular space invasion.

### Radiomics Analysis

A total of 1409 radiomics features were extracted from arterial and portal venous phases, respectively. The ICC interobserver and intraobserver were good, with 0.70–0.93 and 0.85–0.90, respectively.

The radiomics features were reduced and selected in the arterial and portal venous phase images. The radiomics features that did not significantly differ between the groups or did not show significant correlations with CD8 expression were excluded. The remaining 67 radiomics features were further reduced using a LASSO logistic regression model. Finally, the radiomics characteristics were reduced to 10 features (**Supplemental Figures 1A, B**), and the LASSO logistic regression formula was used to obtain the rad-score (**Table 2**). The rad-score was significantly lower (*p <* 0.001) in the CD8-high group (median: -0.43; range: -1.61−1.42) than in the CD8-low group (median: -0.16; range: -1.16−2.35) (**Supplemental Figure 1C**).

**Table 2:** The radiomics features selected by Lasso Regression.

Phase	Prediction model	
Intercept	-0.1905	
	*ß*	Radiomics name
Arterial phase		
	-0.095	exponential_firstorder_Median
	0.028	exponential_firstorder_Variance
	0.0403	square_glszm_SmallAreaLowGrayLevelEmphasis
	-0.0705	wavelet-LHH_firstorder_Mean
	0.0965	wavelet-HLH_glszm_SizeZoneNonUniformity
	-0.1691	wavelet-HLH_glszm_LowGrayLevelZoneEmphasis
	0.2466	wavelet-HHH_firstorder_Mean
	0.1375	lbp-2D_firstorder_Skewness
Portal venous phase		
	-0.1429	wavelet-LLH_glszm_SmallAreaHighGrayLevelEmphasis
	-0.2314	wavelet-HHL_glszmSmallAreaEmphasis

Radiomics score = -0.1905 - 0.095 × exponential_firstorder_Median (Arterial phase).

+ 0.028 × exponential_firstorder_Variance (Arterial phase).

+ 0.0403 × square_glszm_SmallAreaLowGrayLevelEmphasis (Arterial phase).

- 0.0705 × wavelet-LHH_firstorder_Mean(Arterial phase).

+ 0.0965 × wavelet-HLH_glszm_SizeZoneNonUniformity(Arterial phase).

- 0.1691 × wavelet-HLH_glszm_LowGrayLevelZoneEmphasis (Arterial phase).

+ 0.2466 × wavelet-HHH_firstorder_Mean (Arterial phase).

+ 0.1375 × lbp-2D_firstorder_Skewness (Arterial phase).

- 0.1429 × wavelet-LLH_glszm_SmallAreaHighGrayLevelEmphasis (Portal phase).

- 0.2314 × wavelet-HHL_glszmSmallAreaEmphasis (Portal phase).

### Univariate Analysis

The results of the univariate analysis (**Table 3**) demonstrated that the rad-score and T stage were significantly associated with CD8 expression.

**Table 3:** The result of univariate analysis.

Variables	Training set		Validation set	
	OR (95% CI)	*p-* Value	OR (95% CI)	*p-* Value
Rad-score	5.16 (2.10, 12.68)	0.0004	4.99 (1.47, 16.93)	0.01
Sex				
Male	1.0		1.0	
Female	0.77 (0.37, 1.59)	0.48	1.02 (0.32, 3.27)	0.97
Age	1.00 (0.97, 1.04)	0.82	1.01 (0.95, 1.07)	0.80
BMI	1.02 (0.91, 1.15)	0.71	1.00 (0.98, 1.01)	0.70
Operation				
Pancreaticoduodenectomy	1.0		1.0	
Distal pancreatectomy	0.57 (0.29, 1.16)	0.12	0.64 (0.19, 2.20)	0.48
T stage				
T1-2	1.0		1.0	
T3-4	0.34 (0.17, 0.69)	0.0029	0.50 (0.15,1.64)	0.25
N stage				
N0	1.0		1.0	
N1	1.93 (0.93, 4.03)	0.08	1.33 (0.34, 5.19)	0.68
N2	0.36 (0.11, 1.21)	0.10	2.40 (0.55, 10.53)	0.25
Grade of differentiation				
Well-moderately	1.0		1.0	
Poorly-undifferentiated	0.99 (0.47, 2.07)	0.97	2.08 (0.64, 6.74)	0.22
Duodenum Invasion				
Negative	1.0		1.0	
Positive	1.09 (0.53, 2.23)	0.82	1.02 (0.32, 3.27)	1.00
Bile Invasion				
Negative	1.0		1.0	
Positive	1.45 (0.73, 2.90)	0.29	2.47 (0.75, 8.17)	0.14
LVSI				
Negative	1.0		1.0	
Positive	0.60 (0.29, 1.24)	0.17	2.47 (0.75, 8.17)	0.14
Perineural invasion				
Negative	1.0		1.0	
Positive	1.40 (0.32, 6.13)	0.66	1.67 (0.14, 19.76)	0.69
Tumor size (cm, mean ± SD)	0.80 (0.64, 1.01)	0.06	0.66 (0.42, 1.04)	0.07
Location				
Head	1.0		1.0	
Body and tail	0.57 (0.29, 1.16)	0.12	0.64 (0.19, 2.20)	0.48
Parenchymal atrophy				
No	1.0		1.0	
Yes	0.70 (0.35, 1.37)	0.30	0.94 (0.30, 2.98)	0.92
PD cutoff and dilation				
No	1.0		1.0	
Yes	0.93 (0.41, 2.09)	0.86	1.11 (0.31, 3.92)	0.87
CBD cutoff and dilation				
No	1.0		1.0	
Yes	1.20 (0.60, 2.40)	0.60	1.24 (0.39, 3.94)	0.72
Pancreatitis				
No	1.0		1.0	
Yes	1.03 (0.52, 2.03)	0.94	0.62 (0.19, 1.98)	0.42
Contour abnormality				
No	1.0		1.0	
Yes	1.04 (0.38, 2.82)	0.94	1.80 (0.39, 8.27)	0.45
Cyst n (%)				
No	1.0		1.0	
Yes	1.90 (0.51, 7.07)	0.34	0.28 (0.03, 2.67)	0.27
Vascular invasion				
No	1.0		1.0	
Yes	0.75 (0.34, 1.64)	0.47	1.36 (0.39, 4.76)	0.63

OR, odds ratio; CI, confidence interval; Rad-score radiomics score; BMI, body mass index; LVSI, lymphvascular space invasion; PD, pancreatic duct; CBD, common bile duct; Rad-score, radiomics score.

### Development, Performance, and Validation of the Prediction Model

**Figure 4:**
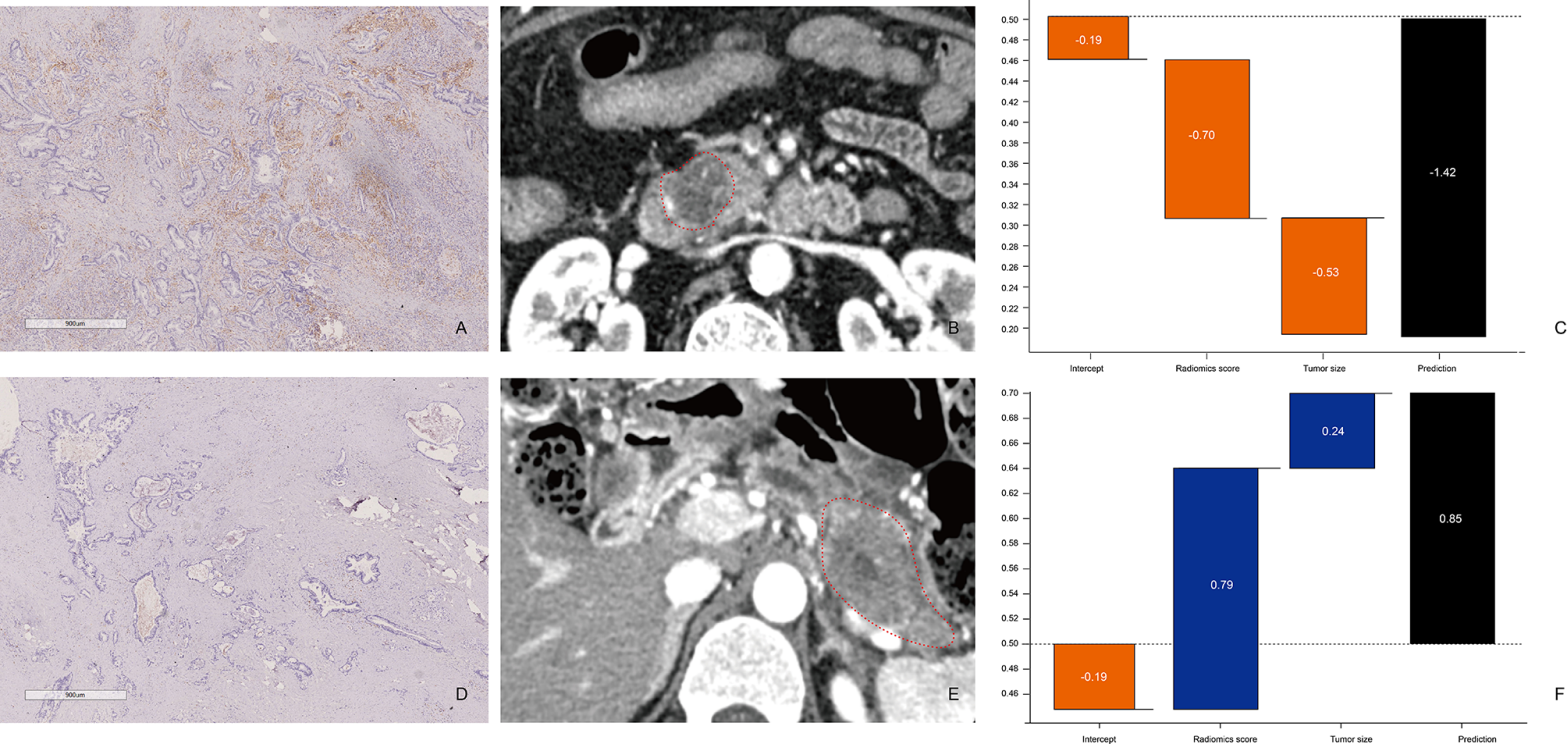
Comparison between patients with low and high CD8^+^ T-cell infiltration **(A–C)** Patient 1: A 65-year-old man with PDAC in the CD8^+^ T-high group. **(A)** CD8^+^ T-cell infiltration is high (×20). **(B)** The axial portal-phase CT image shows an infiltrative, low-attenuation mass (arrows) located at the pancreatic head. **(C)** The prediction probability of low CD8^+^ T infiltration was 80.58% by XGBoost classifier. **(D–F)** Patient 2: A case of a 49-year-old man with PDAC in the CD8^+^ T-low group. **(D)** CD8^+^ T-cell infiltration is low (×20). **(E)** The axial portal-phase CT image shows an infiltrative, low-attenuation mass (arrows) located at the pancreatic body and tail. **(F)** The prediction probability of low CD8^+^ T-cell infiltration is 70.07% by XGBoost classifier.

**Figure 5:**
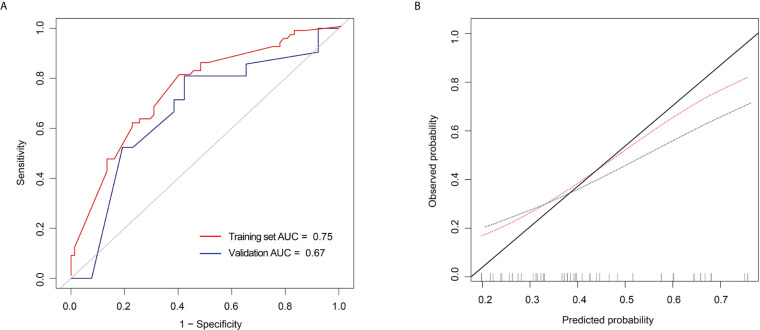
Receiver operating characteristic (ROC) curves and calibration curves of the extreme gradient boosting (XGBoost) classifier **(A)** ROC curves of the XGBoost classifier in the training and validation set. **(B)** Calibration curves of the XGBoost classifier in the training and validation set.

The performance of the prediction model combining radiomics features and tumor size is shown in **Figures 4** and **5**. The AUC values were 0.75 (95% CI: 0.67–0.83) and 0.67 (95% CI: 0.51–0.83) for the training and validation sets, respectively. The sensitivity, specificity, accuracy, positive predictive value, and negative predictive value for the training set were 80.65%, 60.00%, 0.69, 0.63, and 0.79, respectively, whereas those for the validation set were 80.95%, 57.69%, 0.68, 0.61, and 0.79, respectively. The calibration curve showed good calibration of the training (*p* = 0.92) and validation sets (*p* = 0.23).

### Clinical Utility of the Prediction Model

**Figure 6:**
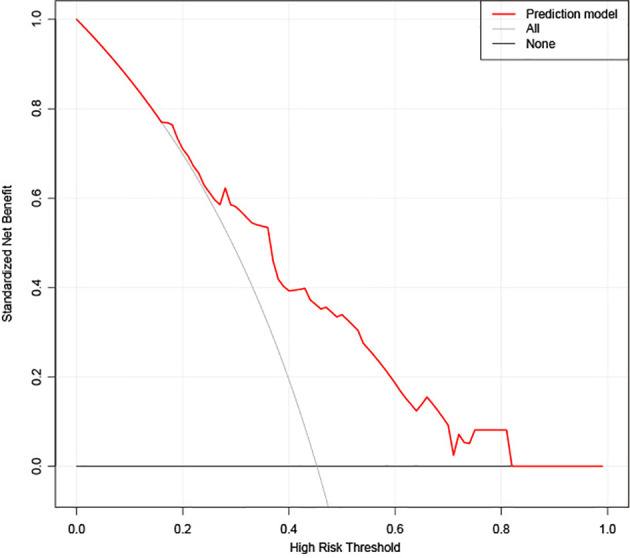
Decision curve analysis (DCA) for the extreme gradient boosting (XGBoost) classifier. The y-axis represents the net benefit. The gray line represents the hypothesis that all patients had high CD8^+^ T-cell infiltration. The black line shows the hypothesis that all patients had low CD8^+^ T-cell infiltration. The x-axis shows the threshold probability, which is where the expected benefit of treatment is equal to the expected benefit of avoiding treatment. The decision curves show that with a threshold probability greater than 0.16, using the prediction model to predict CD8^+^ T-cell infiltration adds more benefit than the treat-all-patients as high CD8^+^ T-cell infiltration scheme or the treat-none as low CD8^+^ T-cell infiltration scheme in the training set.

The decision curve of the rad-score is shown in **Figure 6**. The decision curves show that with a threshold probability >0.16, using the XGBoost classifier to predict CD8^+^ T-cell added more benefit than the “treat all patients as high CD8^+^ T-cell” scheme or the “treat none as low CD8^+^ T-cell” scheme.

## Discussion

Immunotherapy has emerged as a promising treatment in cancer; assessing patients’ different immune statuses with PDAC can better help physicians identify those who can benefit from immune therapies. Although relevant genetic subtypes have been identified (26, 27), clinicians still lack reproducible and biologically meaningful biomarkers to identify patients with favorable prognoses at initial diagnosis. We focused on the radiomic features extracted from the pancreatic protocol CT scan, which is widely used in practice, to identify such a biomarker. Compared with histopathologic and molecular biomarkers, radiomics has the potential to predict the molecular profiles of tumors from image phenotypes inexpensively, non-invasively, and easily. In this study, we observed that the infiltration of CD8^+^ TILs is associated with the prognosis of patients with PDAC. Further, we established a CT-based radiomic score to extrapolate the tumor immune infiltration levels in patients with PDAC.

Cellular immunity is important for the immune system and plays a critical role in eliminating cancer and preventing inflammation. CD8^+^ T-cells can lyse tumor cells directly that expose tumor-specific antigens in various cancers, including PDAC (28). Quantification of CD8^+^ TILs, known as the immunoscore, was developed to evaluate the association between the infiltration level of CD8^+^ TILs and patients with PDAC survival (27, 29–32), with results consistent with those in our study. Our study used X-tile plots (25), a new bioinformatics tool for biomarker assessment and outcome-based cut-point optimization, to provide a global assessment of every possible way of dividing the patients with PDAC into low- and high-level CD8 expression. All patients were divided into either CD8-high (CD8 >18.69%, n = 101; 54.89%) or CD8-low (CD8 ≤18.69%, n=83; 45.10%) groups, based on the optimal cut-off of CD8 level, as determined by x-tile (18.69%). Furthermore, a log-rank test showed that the survival duration in the CD8-high group (22.63 months, 95% CI: 20.20–36.20) was significantly longer than that in the CD8-low group (14.67 months, 95% CI: 12.13–22.37).

Compared to the immunoscore of surgical tissue samples, measuring the level of CD8^+^ TILs by radiomics is more convenient, which is especially important in patients with unresectable PDAC. Sun et al. built a CT-based radiomic signature to assess CD8^+^ TIL infiltration determined by RNA-seq data (33). More than fifteen types of tumors were included in this study, but not PDAC. We are the first to have investigated the possibility of extrapolating the infiltration levels of CD8^+^ TILs in PDAC using radiomics based on CT in both a training and validation cohort. The rad score can reflect the infiltration level of CD8^+^ TIL, and the association between lower rad scores and higher CD8^+^ TIL infiltration can be observed, suggesting that the rad score may be an important prognosis biomarker for patients with PDAC. Furthermore, the DCA test showed that the rad-score could effectively facilitate clinical decision-making.

The intra-tumor heterogeneity assessed by radiomics may reflect genomic heterogeneity, and tumors with more genomic heterogeneity are more likely to resist therapy and develop distant metastasis; thus, they tend to predict a worse prognosis (14, 34–36). Texture analysis is an objective mathematical method based on their gray levels and spatial relationships (37). The most widely used texture analysis methods are the gray-level co-occurrence matrix (GLCM) and gray-level run-length matrix (GLRLM) (38). GLCM can describe the pixel distribution within a region and indicate the frequency of various combinations of grey values observed (38). GLRLM describes the relationships in linear one-dimensional terms (39). Chen et al. observed that highly immune infiltrated HCCs were more homogenous, explaining the high value of GLCM (17). Sun et al. observed that GLRLM could be representative of inflammatory infiltrate, which could reflect homogeneity or heterogeneity of an image (33). In our study, the radiomic signature comprised textural features from the gray-level size-zone matrix (GLSZM). GLSZM is an extended version of GLRLM that describes the size and intensity of voxels clusters in a region of interest (40), which has proven useful when the main characteristic is heterogeneity (40).

There are several limitations to this study. First, our validation cohort was from the same center as the training cohort, which restricts our findings’ generalizability to other centers. Second, as a retrospective single-center study, the relatively small sample size may weaken our conclusion. The sample size should be increased to help draw a more reliable result. Third, a few studies have found the importance of joint analysis of PD-L1 expression with CD8 expression, which may explain the mechanism of the immunosuppressive microenvironment of PDAC (20, 41, 42). However, several studies (20, 41) have observed that the PD-L1^-^/CD8^high^ subtype had the best survival, whereas patients with low CD8 expression had similar survival regardless of PD-L1 status, which means the endogenous CD8^+^ TIL-mediated antitumor immune response may play a key role in the prognosis of patients with PDAC. Therefore, evaluating CD8 infiltration levels should be prioritized in a limited timeframe. Fourth, a few recent studies have suggested that the combination of intratumoral and peritumoral radiomics is more effective in predicting therapeutic outcomes (17, 43). Therefore, in the future, further studies involving peritumoral radiomics in larger populations are needed. In addition, the prediction performance of XGBoost in this study is not fully satisfactory, so we will continue to explore other deep learning models to improve the diagnostic efficiency in the future.

## Conclusion

In conclusion, our study established and validated an enhanced CT-based rad-score for predicting the infiltration level of CD8^+^ TILs in patients with PDAC. This rad-score may be useful in the pretreatment prediction of individual patient immunoscores to guide accurate prognosis prediction and precision immunotherapy for patients with PDAC.

## Data Availability Statement

The original contributions presented in the study are included in the article/**Supplementary Material**. Further inquiries can be directed to the corresponding authors.

## Ethics Statement

The studies involving human participants were reviewed and approved by Biomedical Research Ethics Committee of Changhai hospital. The patients/participants provided their written informed consent to participate in this study. Written informed consent was obtained from the individual(s) for the publication of any potentially identifiable images or data included in this article.

## Author Contributions

Guarantors of the integrity of entire study: YB and CW. Study design or data acquisition or data analysis/interpretation: all authors. Manuscript drafting or manuscript revision: JL, ZS, and FL. Approval of final version of submitted manuscript: all authors. Literature research: XCF, YM, HZ, XF, and QL. Clinical studies: JY, HJ, YL, LW, and CW. Statistical analysis: YB. Manuscript editing: JL and FL. Supervision: YB and JPL. All authors contributed to the article and approved the submitted version.

## Funding

This work was supported in part by the National Science Foundation for Scientists of China (81871352), Clinical Research Plan of SHDC (SHDC2020CR4073), 234 Platform Discipline Consolidation Foundation Project (2019YPT001), and Shanghai Science and Technology Innovation Action Plan Medical Innovation Research Project (20Y11912500).

## Conflict of Interest

The authors declare that the research was conducted in the absence of any commercial or financial relationships that could be construed as a potential conflict of interest.

## Supplementary Material

Supplementary Figure 1 Radiomic feature selection using a parametric method, the least absolute shrinkage and selection operator (LASSO). **(A)** Selection of the tuning parameter (λ) in the LASSO model via 10-fold cross validation based on minimum criteria. Binomial deviances from the LASSO regression cross-validation procedure have been plotted as a function of log (λ). The y-axis indicates binomial deviances. The lower x-axis indicates the log (λ). Numbers along the upper x-axis represent the average number of predictors. Red dots indicate the average deviance values for each model with a given λ, and vertical bars through the red dots show the upper and lower values of the deviances. The vertical black lines define the optimal values of λ, where the model provides its best fit to the data. The optimal λ value of 0.05 with log (λ) = -3.05 has been selected. **(B)** LASSO coefficient profiles of the 67 texture features. The dotted vertical line is plotted at the value selected using 10-fold cross-validation in A. The 10 resulting features with non-zero coefficients are indicated in the plot. **(C)** The error-bar chart of the 10 radiomics features and radiomics score.
