# Supplementary figures and images for: XGBoost Classifier Based on Computed Tomography Radiomics for Prediction of Tumor-Infiltrating CD8+ T-Cells in Patients With Pancreatic Ductal Adenocarcinoma
Source: Front Oncol. 2021 May 19;11:671333. doi: 10.3389/fonc.2021.671333 (PMC8170309; doi:10.3389/fonc.2021.671333)

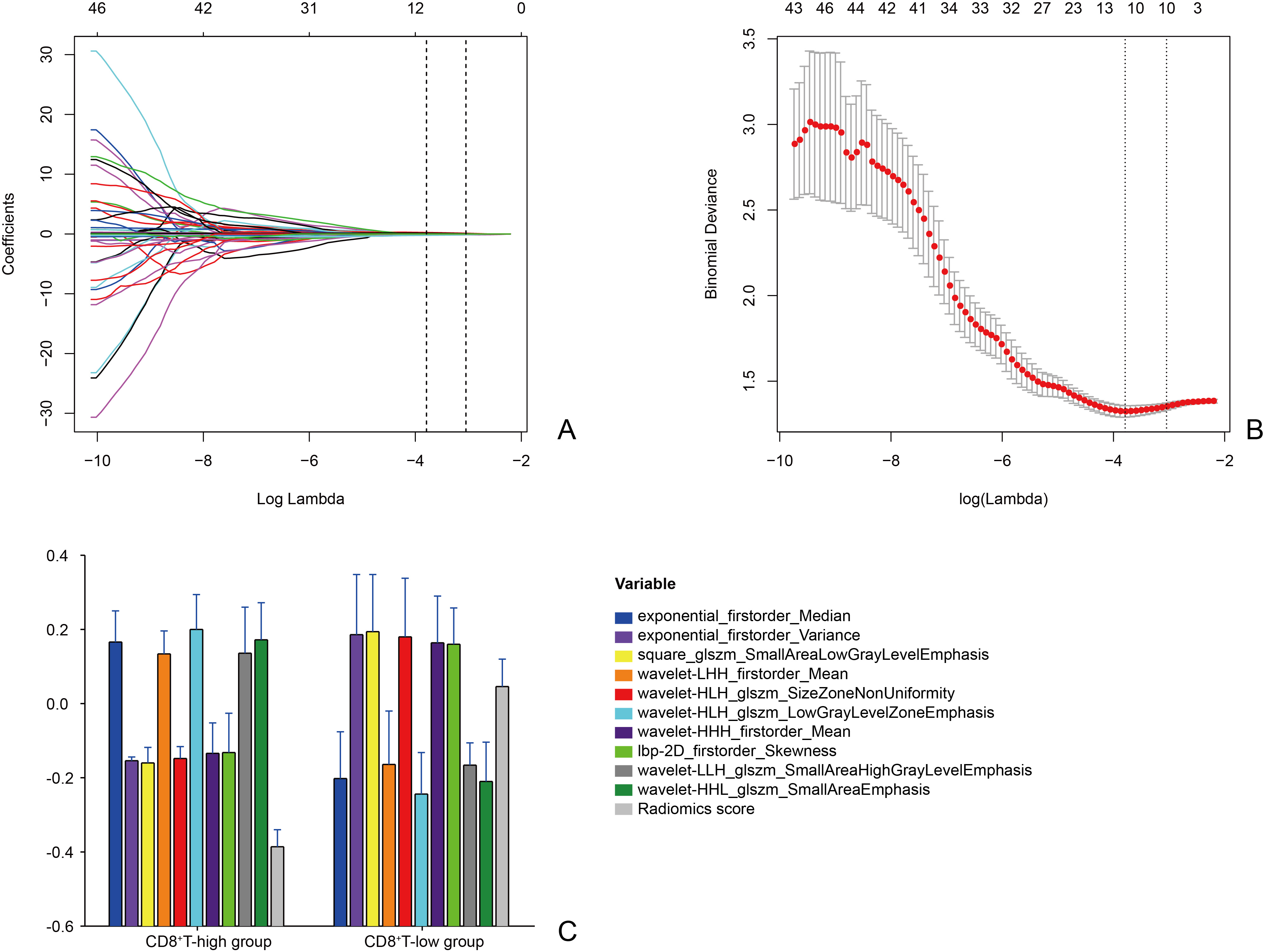

Supplement: Supplementary Figure 1 — Radiomic feature selection using a parametric method, the least absolute shrinkage and selection operator (LASSO). (A) Selection of the tuning parameter (λ) in the LASSO model via 10-fold cross validation based on minimum criteria. Binomial deviances from the LASSO regression cross-validation procedure have been plotted as a function of log (λ). The y-axis indicates binomial deviances. The lower x-axis indicates the log (λ). Numbers along the upper x-axis represent the average number of predictors. Red dots indicate the average deviance values for each model with a given λ, and vertical bars through the red dots show the upper and lower values of the deviances. The vertical black lines define the optimal values of λ, where the model provides its best fit to the data. The optimal λ value of 0.05 with log (λ) = -3.05 has been selected. (B) LASSO coefficient profiles of the 67 texture features. The dotted vertical line is plotted at the value selected using 10-fold cross-validation in A. The 10 resulting features with non-zero coefficients are indicated in the plot. (C) The error-bar chart of the 10 radiomics features and radiomics score. [file Image_1.jpeg]
