# Supplementary material for: XGBoost Classifier Based on Computed Tomography Radiomics for Prediction of Tumor-Infiltrating CD8+ T-Cells in Patients With Pancreatic Ductal Adenocarcinoma
Source: Front Oncol. 2021 May 19;11:671333. doi: 10.3389/fonc.2021.671333 (PMC8170309; doi:10.3389/fonc.2021.671333)
Supplement: Supplementary file 2 [file DataSheet_1.docx]

2.2 CT Scanning

The CT scan parameters were as follows: 120 kV; effective mAs, 150; beam collimation, 160 × 0.5 mm; matrix, 350×350; and gantry rotation time, 0.5 s. A nonenhanced CT scan was performed first, followed by a dynamic contrast-enhanced CT scan. The scan delay time was determined on the basis of the test bolus. The contrast agent (90–95 mL of 355 mgI/mL iopromide; Ultravist 370, Bayer Schering Pharma, Berlin, Germany) was injected at a rate of 5.5 mL/s with a power injector (Medrad Mark V plus, Bayer, Leverkusen, Germany) via the forearm vein, followed by an injection of 98 mL of normal saline to flush the tube. The contrast-enhanced CT scan was performed in arterial (20–25 s), portal venous (60–70 s), and delayed (110–130 s) phases after contrast agent injection. The slice thickness/intervals of the scan were 0.8/1.0 mm, respectively. The scanning range was from the level of the diaphragm to that of the pelvis.

Differences in medical imaging factors can cause inconsistencies in the image intensity information from tissues of the same nature. We used the following formula for intensity normalization (where *x* represents the original intensity; f (*x*) indicates normalized intensity; $\mu$ indicates the average value; $\sigma$refers to variance; and *s* is an optional scaling ratio, which has been set to 1 by default). While retaining the intensity difference of the diagnostic value, the image intensity inconsistency caused by the difference in imaging parameters is reduced or even eliminated for subsequent imaging radiomics analysis.

Equation 1 ([*https://pyradiomics.readthedocs.io/en/latest/radiomics.html#radiomics.imageoperations.normalizeImage*](https://pyradiomics.readthedocs.io/en/latest/radiomics.html#radiomics.imageoperations.normalizeImage))

$$f\left( x \right)=\frac{s (x-u_{x})}{\sigma_{x}}$$

2.4 Radiological Imaging Analysis

We used the original cross-sectional images for the analysis. All the images were analyzed by two abdominal radiologists with 30 and 10 years of experience, respectively. They were blinded to the clinical and pathological details. The final results were determined by consensus.

All tumors were evaluated for the following characteristics: (1) CT-reported tumor size [i.e., the maximum cross-sectional diameter of the tumor[1]]; (2) tumor location: pancreatic head, body, and tail; (3) pancreatitis identified by stranding of the peripancreatic fat tissue, ill-defined parenchymal contours, and fluid collections in the peripancreatic region; (4) pancreatic duct (PD) cut-off and dilation (>3 mm); (5) common bile duct (CBD) cut-off and dilation (>10 mm); (6) parenchymal atrophy; (7) contour abnormality; (8) cyst: presence of any pseudocysts and retention cysts; and (9) vascular invasion: invasion of the common hepatic artery, splenic artery and vein, celiac artery trunk, gastroduodenal artery, superior mesenteric artery and vein, and portal venous vein. The criteria were vessel occlusion, stenosis, or more than half of the perimeter being in contact with the tumor.

2.5 Radiomics Workflow

The details are shown in Appendix 1.We extracted the volume of interest (VOI) for each patient by stacking the corresponding regions of interest (ROIs), delineated slice-by-slice. Radiomics feature extraction was performed using the open-source Python package Pyradiomics 1.2.0 (http://www.radiomics.io/pyradiomics.html)[2]. We used two classes of feature extraction methods: original feature and filter class, which, in turn, included seven categories: logarithm, exponential, gradient, square, square root, lbp-2D, and wavelet. A total of 1409 2D and 3D features from primary tumors in the portal venous phase were extracted and divided into seven groups: (a) first-order statistics, (b) shape features, (c) gray-level co-occurrence matrix (GLCM) features, (d) gray-level dependence matrix (GLDM) features, (e) gray-level run-length matrix (GLRLM) features, (f) gray-level size-zone matrix (GLSZM) features, and (g) neighborhood gray-zone difference matrix (NGTDM) features. Feature selection comprised three steps: variance analysis, Spearman correlation analysis, and LASSO logistic regression algorithm. Finally, a radiomics score (rad-score) was calculated for each patient via a linear combination of selected features that were weighted by their respective coefficients.

Reference

1 Watanabe, H., Okada, M., Kaji, Y., Satouchi, M., Sato, Y., Yamabe, Y., Onaya, H., Endo, M., Sone, M., and Arai, Y. (2009). [New response evaluation criteria in solid tumours-revised RECIST guideline (version 1.1)]. *Gan To Kagaku Ryoho* 36**,** 2495-2501.

2 Van Griethuysen, J.J.M., Fedorov, A., Parmar, C., Hosny, A., Aucoin, N., Narayan, V., Beets-Tan, R.G.H., Fillion-Robin, J.C., Pieper, S., and Aerts, H. (2017). Computational Radiomics System to Decode the Radiographic Phenotype. *Cancer Res* 77**,** e104-e107.
